# Engineering Drought Resistance in Forest Trees

**DOI:** 10.3389/fpls.2018.01875

**Published:** 2019-01-08

**Authors:** Andrea Polle, Shao Liang Chen, Christian Eckert, Antoine Harfouche

**Affiliations:** ^1^Beijing Advanced Innovation Center for Tree Breeding by Molecular Design, College of Biological Sciences and Technology, Beijing Forestry University, Beijing, China; ^2^Forest Botany and Tree Physiology, University of Goettingen, Göttingen, Germany; ^3^Centre of Biodiversity and Sustainable Land Use, University of Goettingen, Göttingen, Germany; ^4^Department for Innovation in Biological, Agrofood and Forest systems, University of Tuscia, Viterbo, Italy

**Keywords:** water limitation, antioxidative systems, genetic engineering, forest tree species, isohydric, anisohydric, avoidance, tolerance

## Abstract

Climatic stresses limit plant growth and productivity. In the past decade, tree improvement programs were mainly focused on yield but it is obvious that enhanced stress resistance is also required. In this review we highlight important drought avoidance and tolerance mechanisms in forest trees. Genomes of economically important trees species with divergent resistance mechanisms can now be exploited to uncover the mechanistic basis of long-term drought adaptation at the whole plant level. Molecular tree physiology indicates that osmotic adjustment, antioxidative defense and increased water use efficiency are important targets for enhanced drought tolerance at the cellular and tissue level. Recent biotechnological approaches focused on overexpression of genes involved in stress sensing and signaling, such as the abscisic acid core pathway, and down-stream transcription factors. By this strategy, a suite of defense systems was recruited, generally enhancing drought and salt stress tolerance under laboratory conditions. However, field studies are still scarce. Under field conditions trees are exposed to combinations of stresses that vary in duration and magnitude. Variable stresses may overrule the positive effect achieved by engineering an individual defense pathway. To assess the usability of distinct modifications, large-scale experimental field studies in different environments are necessary. To optimize the balance between growth and defense, the use of stress-inducible promoters may be useful. Future improvement programs for drought resistance will benefit from a better understanding of the intricate networks that ameliorate molecular and ecological traits of forest trees.

## Introduction

Forests cover about 30% of the terrestrial land (FAO, 2016). They have strong effects on the local climate (Li et al., 2015), by interacting with biogeochemical water cycles (Ellison et al., 2017). When forest trees die or forests are cleared across large-scale landscapes, the negative consequences of drought are aggravated (Allen et al., 2015; Reyer et al., 2015), as shown for many areas world-wide (Laurance, 1998, 2004; van der Werf et al., 2008; Malone, 2017). Over-utilization of forests as a feedstock for energy, construction materials, or the generation of value-added products for the chemical industries, intensifies the problem.

The negative consequences of drought become even more urgent in current times of climate change because projections suggest that such events will occur more frequently and be more extreme (Allen et al., 2010; Reyer et al., 2015). In the past decades, global warming has resulted in a drastic reduction of ice-covered northern polar areas during summer (NSIDC, 2018). Over smaller polar areas air temperatures are cooling down less, thus, resulting in lower differences between boreal, temperate and tropical areas. A possible climate implication of this atmospheric situation is an effect on jet-stream oscillation, which in turn may extend stable meteorological high- and low-pressure (anticyclone/cyclone) conditions; the consequences of such conditions are manifested in periods of precipitation on the one hand, and periods of drought on the other hand (Schaller et al., 2018). During lows, flooding events are frequent, whereas the long-lasting highs lead to scarcity of water in many regions world-wide (FAO, 2016). The dry spells promote salt accumulation in upper soil layers, soil degradation, and erosion (Polle and Chen, 2015). Salt and drought are, thus, often co-occurring stresses with which plants have to cope although their physiological implications vary to some extent (Chen and Polle, 2010; Polle and Chen, 2015). The current review is focused on tree responses and improvement by genetic engineering in response to drought. Since most studies in which trees were ameliorated for improved stress resistance included both drought and salt, salinity cannot be completely ignored.

In this review we highlight the molecular physiology of drought stressed forest trees and present an overview on recent biotechnological approaches to improve the drought tolerance of trees with a focus on yield and enhanced stress resistance. Drought effects on woody plants and measures for tree improvements have regularly been reviewed (Wang et al., 2003; Polle et al., 2006; Rennenberg et al., 2006; Fischer and Polle, 2010; Harfouche et al., 2014). Therefore, this review briefly recapitulates the molecular physiology of drought and salt tolerance mechanisms. We summarize novel studies, published in the past 5 years on the performance of trees engineered for better osmotic resistance. We also pinpoint research gaps that need to be addressed for future improvement of drought resistance in trees.

### Concepts and Strategies

**Figure 1:**
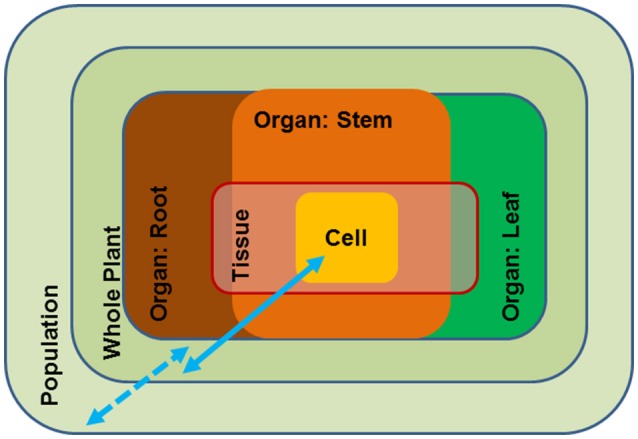
Schematic overview of the different scales of drought impact from the cellular to the population level. Blue arrows: full line (scale of molecular and physiological traits), broken line (scale of ecological traits).

Growth and reproduction of plants requires access to water. Water is the solvent for nutrients in soil, the transport medium for nutrients in the plants and the solvent for cellular solutes. Because water is essentially the “stuff of life”, the plant water status is tightly controlled by a multitude of general and specific measures such as stomatal control on water loss (Buckley, 2005; Daszkowska-Golec and Szarejko, 2013), osmotic adjustment (Harfouche et al., 2014), anatomical adjustment of the water conducting system (Sperry and Love, 2015; Leuschner and Meier, 2018), deposition of cuticular waxes (Hadley and Smith, 1990) and morphological adjustments such as leaf shedding to avoid uncontrolled desiccation (Munné-Bosch and Alegre, 2004; Fischer and Polle, 2010). Periods of severe and long-lasting drought threaten the existence of plants when overruling their acclimation capacities. These broad examples show that drought responses act at different scales, i.e., inside the plant body and at the level of populations; the responses occur at different time scales, and thus, invoke short- and long-term adjustments that can be flexible or reflect evolutionary adaptation (Figure 1). As a result, drought resistance can be achieved by avoidance (homeostasis of tissue water status) or by tolerance mechanisms (acclimation that enable metabolism at a low water potential) (Levitt, 1980; Jones, 1993). These distinctions are important when considering strategies for engineering drought resistance in tree species.

Avoidance mechanisms generally act at the scale of organs or at the whole-plant and the species level (Figure 1). Drought adaptation is characterized by ecological traits such as leaf thickness, root morphology, leaf shedding, etc. It is obvious that these traits are the result of a distinct developmental repertoire in a given species. Due to their presumed complexity they have rarely been incorporated in molecular breeding programs. However, the availability of increasing number of tree reference genomes may open new avenues to better understanding and exploiting their ecological traits. For example, the genomes of European beech (*Fagus sylvatica*) and pedunculate oak (*Quercus robur*) have recently been published (Mishra et al., 2018; Plomion et al., 2018). These two species are closely related members of the *Fagaceae* but exhibit strongly divergent ecological behavior (Aranda et al., 2015; Roman et al., 2015). Beech has a shallow fine root system, while oak has a deep root system (Leuschner and Meier, 2018). Deeper tree roots make a valuable contribution to drought resistance and thus, root morphology is one of the traits targeted for improving water use by capturing subsoil water. At the whole-plant level drought stress avoidance is dependent on the capability of the tree to minimize loss and maximize uptake of water (Chaves et al., 2003) through stomatal control and extensive, deep root systems (Nguyen and Lamant, 1989; Brodribb et al., 2010). We envisage that exploiting genomic information, for instance by comparing the molecular differences in root development of important forest species such as beech and oak, novel approaches that could direct breeding for drought avoidance may become available.

Another interesting example for drought avoidance is leaf shedding, a common phenomenon in tropical dry forests (Wolfe et al., 2016). Leaf shedding is controlled by an intricate interplay of phytohormones, including ethylene, abscisic acid (ABA), and auxin (González-Carranza et al., 1998; Chen et al., 2002a,b; Jin et al., 2015; Paul et al., 2018), which could be harnessed to improve tree drought resistance. In polyploid poplars regenerated from protoplast fusion accelerated drought-induced leaf shedding was observed that resulted in increased tree survival under extreme drought (Hennig et al., 2015). The exact genetic basis of this phenotype is not known but it is apparently associated with partial genome duplication (Hennig, 2016).

Trees must exist in their environment over decades and centuries and therefore require not only drought adaptedness but also metabolic flexibility to adjust their metabolism to changing conditions. Drought tolerance is usually achieved by biochemical modification of the cellular metabolism (Figure 1). Acclimation to drought by an individual plant invokes changes in membrane composition, protection of protein folding, osmotic adjustment, scavenging of reactive oxygen species (ROS), etc. (Harfouche et al., 2014) and, thus, acts at the level of cells to organs (Figure 1). An important feature of plant drought tolerance is the increase in osmotic pressure as a countermeasure to maintain water flux under declining soil water potentials. The production of osmolytes is costly in terms of carbohydrates because it diverts carbohydrates from growth to defense. A striking example of how woody species from arid, saline deserts can economize their carbon budget is the succulent xerophyte *Zygophyllum xanthoxylum* (Janz and Polle, 2012). This species exploits sodium as a “cheap” osmolyte, thereby, improving photosynthesis and growth under harsh environmental conditions (Ma et al., 2012). The discovery of such amelioration mechanisms constitutes an important basis to improve drought tolerance in trees (Bao et al., 2015, more details are found below) and underpins our understanding of the physiological consequences of novel features, which is crucial to harness the critical molecular mechanisms for drought acclimation and adaptation.

## Molecular Physiology of Osmotic Stresses

### Roots

Roots are the first organ to sense and signal soil water deficits (Hamanishi and Campbell, 2011; Brunner et al., 2015). Since enhanced salinity decreases water availability to roots by increasing the osmotic potential in soil solution, the consequences for water uptake are partly similar to those of drought. Both drought and salt result in a decline in root-to-shoot water flow in poplars (Chen et al., 1997, 2002b; Shi et al., 2010), but the consequences are generally less severe in salt tolerant than in sensitive species (Chen et al., 2002b,c, 2003).

**Figure 2:**
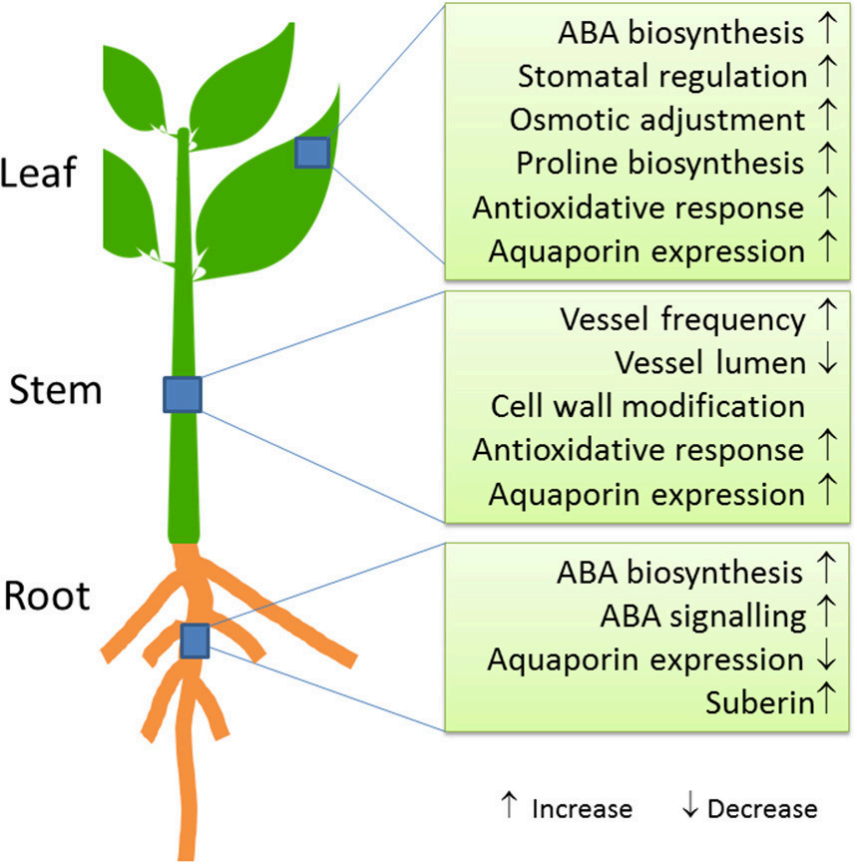
Molecular and physiological key responses of trees to drought stress.

At the biochemical level, increased ABA concentrations are a hallmark of osmotic stress across all organs (Wasilewska et al., 2008; Kuromori et al., 2018) (Figure 2). The stress signal ABA interacts with pyrabactin resistance 1 (PYR1)/PYR1-like (PYL)/regulatory components of ABA receptors (RCAR) proteins, which then can then form a complex with PP2Cs (Type 2C phosphatases). Thereby, phosphorylation of a SnRK (Kinase) is enabled, which subsequently activates down-stream transcription factors and target genes (Fujita et al., 2011; de Zelicourt et al., 2016). Transcriptomic analyses of pine and poplar roots under drought revealed upregulation of genes for ABA biosynthesis [9-cis-epoxycarotenoid dioxygenase (*NCED*)], signaling and response factors such as *DREB1, bZIP, AP2/ERF, MYB, NAC*, and *WRKY* (Wilkins et al., 2009; Cohen et al., 2010; Lorenz et al., 2011; Perdiguero et al., 2012). Salinity and drought share similar response patterns in poplar roots, which are likely mediated by ABA (Chen et al., 1997, 2001, 2002b; Luo et al., 2009).

ABA in roots further promotes increased biosynthesis of proline (Davies and Bacon, 2003). High concentrations of proline may act as osmolyte and contribute to osmotic adjustment; a further possible function of proline is the protection of appropriate protein folding (Claeys and Inzé, 2013). However, only a few studies have demonstrated increases in proline concentrations in the roots of trees in response to drought stress (Cocozza et al., 2010; Naser et al., 2010). This casts doubts on a prominent role of proline as an osmolyte in roots. Another function of ABA biosynthesis and transport in roots may be related to stimulate endodermal suberization (Tan et al., 2003; Koiwai et al., 2004; Kuromori et al., 2010; Kanno et al., 2012; Zhang et al., 2014). A recent study demonstrated that endodermis is reversibly impregnated with hydrophobic compounds such as suberin in response to abiotic stresses, which might restrict apoplastic movement of water (Barberon et al., 2016).

Evidence is accumulating that ABA plays a role in regulating hydraulic conductance in roots possibly via aquaporin activity (Parent et al., 2009; Almeida-Rodriguez et al., 2011). At the molecular level, aquaporins are important for the control of water uptake (Fox et al., 2017). Since contrasting responses to different osmotic stress factors have been observed, their regulation is still unclear. For example, changes in the evaporative demand resulted in aquaporin upregulation (Parent et al., 2009; Almeida-Rodriguez et al., 2011), while drought or salt stress caused declines in their expression (Bogeat-Triboulot et al., 2007; Wang et al., 2017a). A decrease in aquaporins in roots is supposed to increase cellular water conservation due to reduced membrane water permeability during periods of dehydration stress (Smart et al., 2001; Bogeat-Triboulot et al., 2007) and would fit with the observed restriction of apoplastic water loss by enhanced suberization (Barberon et al., 2016).

Mycorrhizal fungi also increase tree stress tolerance by regulation of aquaporins and stress metabolites (Luo et al., 2009; Dietz et al., 2011; Xu et al., 2015; Peter et al., 2016), but an in-depth treatment of this aspect is beyond the scope of this review. The reader is advised to consult Brunner et al. (2015).

### Stem

Physiological responses of trees to drought stress lead to hydraulic and carbon cycle adjustments (Parker, 1956; Bréda et al., 2006). The hydraulic architecture of the stem is important to keep up water transport under drought and re-establish water flux after re-irrigation. Hydraulic acclimation can be achieved by increased vessel frequencies and decreased vessel lumina (Hacke et al., 2006). Drought-resistant trees decrease the ratio of vessel lumen to cell wall thickness to enhance wall strength under water stress (Hacke et al., 2001) (Figure 2). While the anatomy and biophysics of xylem adjustment to water-limited conditions has often been studied (Tyree and Ewers, 1991; Anderegg, 2015; Sperry and Love, 2015), our knowledge on the molecular processes underlying these changes is limited. Aquaporins (PIP1 family) are important for refilling of embolized vessels and thereby helping the tree to recover after drought (Secchi and Zwieniecki, 2011; Laur and Hacke, 2014).

Anatomical and transcriptomic analyses of the developing xylem of poplars revealed that drought imposed changes that are similar to those found after salt exposure (Junghans et al., 2006; Bogeat-Triboulot et al., 2007; Janz et al., 2012; Wildhagen et al., 2018). For example, salt stressed poplar trees reinforce cell walls by increasing wall thickness relative to lumen area and avoid a strong loss in hydraulic conductivity by enhancing vessel frequency (Janz et al., 2012). Transcript abundances of of genes encoding fasciclin-like arabinogalactans (FLA), COBRA-like proteins, xyloglucan-endo-transglycolyases, pectin methylesterases were jointly repressed in developing xylem, while those of genes activating stress and defense responses increased (Janz et al., 2012). Similarly, in water-stressed poplars, transcript abundances of several cellulose synthases, arabinogalactan (AGP) and fasciclin-like proteins decreased (Berta et al., 2010). Wildhagen et al. (2018) also reported massive changes in the regulation of genes required for cell wall forming enzymes. Unexpectedly, drought decreased lignin and increased the saccharification potential of the wood (Wildhagen et al., 2018), indicating positive changes with regard to the biotechnological usage of wood. It would therefore be worthwhile to test whether these changes can be achieved without the typical drought-induced growth-defense trade-off.

Drought further activates antioxidant defenses in the cambium of different poplar clones [Dvina (*P. deltoides*), I-214 (*P*. × *canadensis*), Pallara et al., 2012]. A distinct isoform of CATALASE 3 (CAT3) was strongly enhanced under water deficit, unpinning an essential role for this enzyme in ROS control under drought stress (Pallara et al., 2012). Furthermore, increases in the concentrations of osmotically active solutes in the cambial region of *P. alba* accompanied reductions in predawn leaf water potentials and stem dehydration (Pallara et al., 2012).

### Leaves

Stomatal regulation is one of the most important mechanisms to adjust water consumption to fluctuations in water availability (Tardieu and Simonneau, 1998; McDowell et al., 2008; Skelton et al., 2015). Regulation of stomatal aperture reduces water loss by leaf transpiration (Stålfelt, 1955; Barrs, 1971; Brodribb and Holbrook, 2003; Araújo et al., 2011) but there is a trade-off between transpirational water loss and CO_2_ assimilation (Jarvis and Jarvis, 1963; Cowan, 1978). Pioneering studies involving poplar species and hybrids shed light on two drought stress response strategies, anisohydric and isohydric behavior with divergent consequences for water flux and biomass production (Marron et al., 2003; Monclus et al., 2006; Giovannelli et al., 2007). Anisohydric plants keep their stomata relatively widely open and prevent dehydration by increasing the osmotic pressure in leaves (Gebre et al., 1994; Marron et al., 2002; Hanin et al., 2011; Barchet et al., 2014; Martorell et al., 2015); thereby, they are capable to support growth and biomass production (Passioura, 2002). Isohydric plants limit water loss through sensitive stomatal regulation and closure and/or by leaf abscission (Couso and Fernández, 2012). Poplars are isohydric species but exhibit a suite of adaptive measures (Brignolas et al., 2000) such as variation in stomatal sensitivity (Hamanishi et al., 2012), leaf shedding (Marron et al., 2002) and growth decline (Giovannelli et al., 2007). In a population of tree species a continuum of responses to water deficit between isohydryic and anisohydric behavior can be found (Klein, 2014). For instance, beech generally exhibits isohydric behavior but progenies from dry habitats showed stronger anisohydric behavior than those from wet habitats (Nguyen et al., 2017). In poplar, heritability of stomatal responsiveness to water deficit is generally high, indicating that this trait is a useful target for genetic engineering (Orlovic et al., 1998; Al Afas et al., 2006; Monclus et al., 2006).

Endogenous ABA is rapidly produced upon water deficit, initiating a signaling cascade that results in down-stream responses such as stomatal closure (Fujii et al., 2009). Besides roots, ABA biosynthesis takes place in leaves (Kuromori et al., 2018). Stomatal responsiveness to ABA shows large variation among different species and is evolutionary determined (Sussmilch et al., 2017). In angiosperms, ABA induced stomatal closure is usually rapid and can occur within seconds or minutes (Geiger et al., 2011), thus, not requiring *de novo* transcription. Over-expression of the ABA biosynthesis protein 9-cis-epoxycarotenoid dioxygenase 3 (NCED3) is beneficial for water-use efficiency (WUE) and results in enhanced drought resistance in several plant species (Iuchi et al., 2001; Tung et al., 2008). Drought induced changes in stomatal development involve regulation of transcript abundance of the poplar orthologs of *STOMAGEN, ERECTA*, and STOMATA DENSITY AND DISTRIBUTION 1 (*SDD1*) (Harfouche et al., 2014). Interestingly, WUE was increased by the over-expression of a poplar ortholog of *ERECTA* in *A. thaliana* (Xing et al., 2011). ERECTA controls stomatal density but the sequence of events leading to this effect is still unknown (Xing et al., 2011). Genes such as *ERECTA, SDD1*, or *NCED3* should be the focus of future research programs aimed at developing transgenic or gene-edited trees with resistance to naturally occurring field drought conditions.

At the cellular level, biochemical protection measures are activated in response to drought to avoid negative consequences of stress-induced ROS and to endure water deficit (Wang et al., 2003, Figure 2). Moderate water stress results in increased concentrations of soluble carbohydrates and polyols, which potentially promote maintenance of cell turgor in *P. euphratica* leaves through increased osmotic pressure (Bogeat-Triboulot et al., 2007). However, after salt acclimation bulk soluble carbohydrates (including glucose and fructose, sucrose), sugar alcohols, organic acids, mostly decrease or remain almost unaffected (Ottow et al., 2005; Dluzniewska et al., 2007; Ehlting et al., 2007; Brinker et al., 2010), suggesting that moderate salt accumulation in leaves may compensate for osmotic adjustment as observed in some halophytic species (Ma et al., 2012). It is noteworthy that amino acids, in particular proline, increase drastically in both water- and salt-stressed leaves (Brosché et al., 2005; Ottow et al., 2005; Dluzniewska et al., 2007; Ehlting et al., 2007; Pallara et al., 2012). In accordance, the mRNA levels of genes encoding enzymes that catalyze rate-limiting steps of proline synthesis and degradation [delta-1-pyrroline-5-carboxylate synthase (Pc*P5CS*) and proline dehydrogenase] accumulate under osmotic stress (Dluzniewska et al., 2007). However, the bulk rise of proline to μM levels is insufficient to explain the observed change in osmotic pressure in salt exposed trees required to maintain water uptake (Ottow et al., 2005; Brinker et al., 2010). Therefore, increased proline may act as a protectant of protein integrity rather than function as an osmolyte in leaves.

Antioxidative systems also play an important role in the defense against negative consequences of drought stress (Hasanuzzaman et al., 2013). Therefore, one would expect that populations of wild tree species (beech) from dry conditions contain higher ROS protection than those from mesic conditions. Unexpectedly, the opposite was observed: unstressed beech from a mesic habitat showed a higher antioxidative capacity than those from a dry habitat and moreover, those from mesic conditions showed a stronger antioxidative response to drought than those from the dry habitat (Carsjens et al., 2014). These observations suggest that trees exposed infrequently to stress respond more flexibly, whereas long-term stress adapted trees are protected by resistance measures, which are already in place before the onset of acute stress. This view is also supported by constitutively enhanced salt tolerance of *P. euphratica* compared to salt sensitive poplars (Janz et al., 2010). The enhanced tolerance of *P. euphratica* is, for example, based on the expansion of the sodium:proton antiporter family in the genome of this species (Ma et al., 2013). These few and selective examples highlight that divergent strategies may be required for improving drought resistance in short- or long-term water limited environments.

## Genetic Approaches for Increased Stress Tolerance

Because drought and other osmotic stresses result in multiple tolerance or avoidance mechanisms, simple strategies for improving the performance of trees in water-limited environments do not exist. In order to target a suite of genes that can enhance drought tolerance, recent attempts to improve plant performance have often focused on signal perception and transduction (Table 1), whereas overexpression of structural genes found fewer applications (Table 2). Strategies for the selection of candidate genes relied mainly on the inducibility of genes under stress or on the gene origin in a highly stress tolerant species (Tables 1, 2).

**Table 1:** Functional characterization of drought- and salt-inducible protein kinase and transcription factors originating from or expressed in trees species.

**Genes**	**Sources**	**Abiotic and chemical elicitors**	**Functional characterization**				
			**Promoter**	**Transgenic species**	**Phenotypes of stress tolerance**	**Functional traits of transgenics under stress conditions**	**References**
**SIGNAL PERCEPTION AND TRANSDUCTION**							
***PYR*****/*****PYL*****/*****RCAR** PYRL1 PYRL5*	*Populus trichocarpa*	drought ABA	2x CaMV 35S	*Populus davidiana* x *P. bolleana*	Drought osmotic stress cold	↑ stomatal closure	Yu J. et al., 2017
						↑ drought tolerance	
						↑ cold tolerance	
						↑ osmotic stress resistance	
						↓ water loss	
***PYR*****/*****PYL*****/*****RCAR** PYRL1 PYRL5*	*Populus trichocarpa*	drought ABA	2x CaMV 35S	*A. thaliana*	drought	↑ interaction with PP2C	Yu J. et al., 2016
						↑ ABA sensitivity	
						↑ recovery after re-watering	
						↓ seed germination	
***PP2C** HAB1*	*Populus euphratica*	drought ABA	CaMV 35S	*A. thaliana*	drought	↑ ABA response	Chen et al., 2015
						↑ drought sensitivity	
						↑ interaction with PYL4 in yeast two-hybrid assays	
***MAPK** MAPK1*	*Morus L*	Drought Cold Heat NaCl	CaMV 35S	*A. thaliana*	NaCl H_2_O_2_	↑ NaCl tolerance	Liu et al., 2017
						↑ H_2_O_2_ tolerance	
						↓ heat and drought tolerance	
***MYB*** MYB1	*Fraxinus velutina*	NaCl drought	CaMV 35S	*Nicotiana tabacum*	NaCl	↑ biomass production	Li et al., 2016
						↑ SOD and POD activity	
						↑ proline content	
***NAC** NAC034 NAC035 NAC045*	*Populus euphratica*	Drought NaCl	CaMV 35S	*A. thaliana P. tomentosa*	drought NaCl	↑ root development	Lu et al., 2017
						↑ stem elongation	
						↑ drought sensitivity	
						↑ NaCl sensitivity	
**NAC** *NAC3*	*Cicer arietinum*	drought ABA NaCl	CaMV 35S	*Populus deltoides* x *P. euramericana*	drought NaCl	↑ improved growth under drought, comparable to WT	Movahedi et al., 2015
						↑ proline content	
						↑ photosynthetic pigments	
						↑ antioxidant enzymes	
**NAC** *NAC13*	*Tamarix hispida*	NaCl drought	CaMV 35S	*Tamarix hispida A. thaliana*	NaCl osmotic stress	↑ POD and SOD activity	Wang L. et al., 2017
						↑ proline content	
						↑ chlorophyll content	
						↓ ROS	
						↓ MDA	
**NAC** *SNAC1*	*Phyllostachys edulis*	auxin	CaMV 35S	*A. thaliana*	drought PEG	↑ survival rate under drought	Wang L. et al., 2016
						↑ overall growth	
**ErbB-3 binding protein1** *EBP1*	*Hevea brasiliensis*	cold drought ABA	CaMV 35S	*A. thaliana*	drought	↑ enhanced expression of RD29A, RD22 and CYCD3;1	Cheng et al., 2016
						↑ organ size	
**ZFP (zink finger protein)** *ZxZF*	*Zygophyllum xanthoxylum*	drought	RD29A	*thaliana Populus* x *euramericana*	Mannitol ABA	↑ PSII activity	Chu et al., 2016
						↑ root growth	
						↑ antioxidant enzymes	
						↓ H_2_O_2_	
**EPF (epidermal patterning factor)** *PdEPF2*	*Populus nigra* x (*P. deltoids* x *P. nigra*)	drought	CaMV 35S	*A. thaliana*	drought	↑ germination rate	Liu et al., 2016
						↑ primary root length	
						↑ proline content	
						↑ chlorophyll content	
						↑ photosynthetic activity	
**CBF (C-repeat binding factor)** *CBF4*	*Populus euphratica*	cold oxidative stress drought ABA NaCl	CaMV 35S	*Populus tomentosa*	drought ABA NaCl	↑ iWUE	Tian et al., 2017
						↑ photosynthesis rate	
						↑ antioxidative enzymes	
						↑ proline	
						↓ overall growth (dwarf)	
**ERF** *ERF76*	*P. simonii* x *P. nigra*	NaCl	CaMV 35S	*P. simonii* x *P. nigra*	NaCl	↑ ABA	Yao et al., 2016
						↑ GA	
						↑ upregulation of 16 TFs and 45 stress related genes	
**START transcription factor** *EDT1/HDG11*	*A. thaliana*	drought NaCl	CaMV 35S TUB2	*Populus tomentosa, Gossypium hirsutum*	drought NaCl	↑ proline content	Yu L. H. et al., 2016
						↑ soluble sugar content	
						↑ antioxidant enzymes	
						↑ NaCl tolerance	
						↑ drought tolerance	
**CDPK (Calcium -dependent protein kinase)** *PeCPK10*	*Populus euphratica*	Drought Salt Cold	CaMV 35S	*A. thaliana*	Drought Freezing	↑ expression of stress/ABA- responsive genes	Chen et al., 2013
**bZIP (Basic leucine zipper protein)** *ThbZIP1*	*Tamarix hispida*	NaCl PEG 6000 NaHCO_3_ CdCl_2_	CaMV 35S	*Nicotiana tabacum*	Salt	↑ activity of POD and SOD	Wang et al., 2010
						↑ soluble sugars	
						↑ soluble proteins	
**Nucleoside diphosphate kinases** *NDPK2*	*Populus trichocarpa*	ROS	CaMV 35S	*Populus deltoides x P. euramericana*	NaCl drought	↑ auxin related genes	Zhang et al., 2017
						↑ drought tolerance	
						↑ NaCl tolerance	
**Nuclear factor Y** *NF-YB3*	*Picea wilsonii*	NaCl heat PEG not ABA not cold	CaMV 35S	*A. thaliana*	drought NaCl ABA	↑ NaCl tolerance	Zhang et al., 2015
						↑ SOS3 transcript levels	
						↑ drought tolerance	
						↑ CDPK1 transcript levels	
						↑ CBF marker genes	
**DREB (Dehydration -responsive element binding protein)** *PeDREB2a*	*Populus euphratica*	Drought NaCl Low temperature NAA 6-BA GA_3_ Not by ABA	RD29A	*A. thaliana Lotus corniculatus*	Salt Drought	↑ root length and plant height ↑ soluble sugars levels ↓ MDA levels	Zhou et al., 2012
**DREB** *PeDREB2*	*Populus euphratica*	Drought High salinity Cold Not by ABA	CaMV 35S	*Tobacco*	High-salt	↑ seed germination ↑ chlorophyll levels ↑ chlorophyll fluorescence ↓ growth retardation	Chen et al., 2009
**DREB** *PeDREB2L*	*Populus euphratica*	dehydration salt ABA	CaMV 35S	*A. thaliana*	Drought Freezing	↑ DRE/CRT-containing stress- responsive genes, RD29A and RD29B	Chen et al., 2011
**DREB** *ThDREB*	*Tamarix hispida*	NaCl PEG NaHCO_3_ CdCl_2_	CAMV 35S	*Tobacco Tamarix hispida* (transient transgenic)	NaCl Mannitol	↑ antioxidase activity (SOD,POD) ↓ ROS (H_2_O_2_) ↓ MDA content ↓ electrolyte leakage (EL)	Yang et al., 2017
**DREB** *DREB4A*	*Morus alba*	cold drought	CaMV 35S	*A. thaliana N. tabacum*	Drought NaCl heat	↑ regulation of ABI1 and ABI2	Liu et al., 2015
						↑ proline content	
						↓ MDA	
						↑ NaCl, heat and droughttolerance in tobacco	
**DREB** *DREB2A*	*Fraxinus pennsylvanica*	drought	2x CaMV 35S	*Robinia pseudoacacia*	drought	↑ stress-inducible genes	Xiu et al., 2016
						↑ ABA levels	
						↑ auxin levels	
						↓ GA levels	
						↓ zeatin riboside	
**CBL** *PeCBL6 PeCBL10*	*Populus euphratica*	Drought High salinity Cold Not by ABA	CAMV35S	*A. thaliana*	Salinity Drought Low temperature	↑ fresh mass, survival rate ↑ chlorophyll content ↑ chlorophyll fluorescence (Fv/Fm)	Li et al., 2013
**NAC** [No apical meristem (NAM), (CUC)] superfamily *PeNAC1*	*Populus euphratica*	Drought Salt stress ABA (slightly induced)	CaMV 35S	*A. thaliana*	Salt	↑ survival rates, fresh weights	Wang et al., 2013
						↑ capacity for K^+^ uptake and transport	
						↓ AtHKT1 gene expression	
						↓ Na^+^ content	
**NAC** *PeNAC036*	*Populus euphratica*	Drought Salt ABA	CaMV 35S	*A. thaliana* (WT) and mutant *anac072*	Salt Drought	↑ plant height, primary root length (1/2 MS agar medium)	Lu et al., 2017
						↑ survival rate (Soil culture)	
						↑ expression levels of COR47, RD29B, ERD11,RD22 and DREB2A	
**ERF** (Ethylene) response factor *ERF76*	*Populus simonii* x *Populus nigra*	Drought Salinity ABA .	CaMV 35S	*P. simonii* x *P. nigra*	Salt	↑ Plant height, root length, and fresh weight	Wang et al., 2014 Yao et al., 2016
						↑ gene expression of ABA and GA signal pathways	
						↑ defense-related genes, such as LEA, GST and HRGP genes	
						↑ signal transfer-related Genes (incl). P-tyrosine phosphatases, MAPKKK and PR5K	
**WRKY** *ThWRKY4*	*Tamarix hispida*	Drought Salt ABA	CaMV 35S	*A. thaliana*	ABA Salt Oxidative stress	↑ SOD and POD activity	Zheng et al., 2013
						↓ ROS level	
						↓ cell death	
**ZFP** (Zinc finger protein) *ThZFP1*	*Tamarix hispida*	NaCl Mannitol ABA	CaMV 35S	*A. thaliana Tamarix hispida* (transient overexpression or knockdown)	ABA NaCl Mannitol	↑ SOD and POD activity and encoding genes	Zang et al., 2015
						↑ proline level, and *ThP5CS1*&*2* genes	
						↑ chlorophyll content	
						↓ ${\text{O}}_{2}^{-}$ and H_2_O_2_	
						↓ MDA content, membrane lipid peroxidation, electrolytic leakage	

*Polyethyleneglycol (PEG), 1-naphthaleneacetic acid (NAA), 6-benzyl aminopurine (6-BA), gibberellic acid (GA), abscisic acid (ABA), superoxide dismutase (SOD), peroxidase (POD), malondialdehyde (MDA), Cauliflower mosaic virus (CaMV), Electrolyte leakage (EL), instantaneous water use efficieny (iWUE), ↑, Increase; ↓, Decrease*.

*Data were searched between 2013 and 2018 with the key words: Tree and overexpression and drought in Web of Science*.

**Table 2:** Functional characterization of drought- and salt-inducible structural genes originating from or expressed in trees species.

**Genes**	**Sources**	**Abiotic and chemical elicitors**	**Functional characterization**				
			**Promoter**	**Transgenic species**	**Phenotypes of stress tolerance**	**Functional traits of transgenics under stress conditions**	**References**
**DEFENSE AND OTHER STRUCTURAL GENES**							
**Cysteine protease** *SmCP*	*Salix matsudana*	NaCl	CaMV 35S	*A. thaliana*	NaCl	↑ germination rate ↑ SOD activity ↓ MDA content↓ electrolytic leakage	Zheng et al., 2018
**Aquaporin** *PeAQUA1*	*Populus* x *euramericana*	drought NaCl wounding	CaMV 35S	*Populus alba*	zinc	↑ growth rate ↑ intrinsic Transpiration Efficiency	Ariani et al., 2016
**NXH (proton-sodium antiporter)** *ZxNHX **H**^**+**^**-PPase** ZxVP1-1*	*Zygophyllum xanthoxylum*	NaCl drought auxin	CaMV 35S	*Medicago sativa* (Alfalfa)	drought	↑ biomass production ↑ Na^+^, K^+^ and Ca^2+^accumulation in leaves and roots ↑ leaf relative water content ↑ greater photosynthesis capacity	Bao et al., 2015
**Dehydrin** *HbDHN1 HbDHN2*	*Hevea brasiliensis*	ABA ET JA NaCl drought heat wounding	CaMV 35S	*A. thaliana*	NaCl drought water stress osmotic stress	↑ APX & SOD ↑ drought tolerance ↑ NaCl tolerance ↑ tolerance to osmotic stress ↓ H_2_O_2_	Cao Y. et al., 2017
**Ascorbate Peroxidase** *APX*	*Populus tomentosa*	H_2_O_2_	CaMV 35S	*Nicotiana tabacum*	Drought NaCl Oxidative stress	↑ chlorophyll content ↑ NADP to NADPH ratio ↓ H_2_O_2_ ↓ MDA	Cao S. et al., 2017
**XTH** *DkXTH1*	*Diospyros kaki*	darkness cold/heat-shock ET ABA GA IAA	CaMV 35S	*A. thaliana, Solanum lycopersicum*	NaCl drought	↑ NaCl tolerance in A. thaliana and tomato↑ drought tolerance in A. thaliana and tomato	Han et al., 2017
**Choline oxidase** *codA*	*bacteria*	cold NaCl drought	SWPA2 (oxidative stress inducible)	*Populus alba* x *glandulosa*	NaCl drought	↑ higher glycin betain levels ↑ auxin responsive genes ↑ PSII activity ↓ membrane leakage ↓ ROS production	Ke et al., 2016
**FMO (flavin monooxygenase-like)** *YUCCA6*	*A. thaliana*	auxin	SWPA2 (oxidative stress inducible)	*Populus alba* x *glandulosa*	drought	↑ auxin production↑ drought tolerance ↑ PSII activity ↓ membrane leakage ↓ main root growth ↓ membrane leakage	Ke et al., 2015
**GS (glutamine synthase)** *GS1*	*Pinus sp*.	drought nitrogen	CaMV 35S	*Hybrid poplar*	drought	↑ WUE ↑ NUE ↑ glutamine, GABA, putrescin, hydroxyrpoline	Molina-Rueda and Kirby, 2015
**STS (Stilbene synthase)** *MaSTS1 MaSTS2 MaSTS3 MaSTS4*	*Morus atropurpurea*	SA ABA wounding NaCl	CaMV 35S	*Nicotiana tabacum*	heat NaCl PEG	↑ trans-resveratrol levels ↑ drought tolerance↑ NaCl tolerance	Wang et al., 2017b
***CYP450** CYP714A3*	*Populus trichocarpa*	GA	Eui (GA mutant)	*Oryza sativa*	NaCl	↑ NaCl tolerance ↓ GA levels ↓ excessive shoot growth of *eui* mutant is compensated	Wang C. et al., 2016
**Aquaporin** *TIP4;1-1*	*Phyllostachys edullis*	drought NaCl	CaMV 35S	*A. thaliana*	drought NaCl	↑ antioxidant enzymes	Sun et al., 2017
						↑ photosynthetis	
						↓ MDA	
**UDP-galactose-4-epimerase** *PeUGE*	*Phyllostachys edullis*	drought NaCl Water stress	CaMV 35S	*A. thaliana*	Drought NaCl	↑ chlorophyll fluorescence	Sun et al., 2016
**Vacuolar H**^**+**^**-pyrophosphatase** *AVP1*	*Populus trichocarpa*	NaCl drought pH	CaMV 35S	*A. thaliana Populus davidiana × bolleana*	NaCl	↑ prevents NaCl accumulation↑ higher ion efflux	Yang et al., 2015
**Galactinol synthase** *GolS2* **SNF1-related protein kinase** *SRK2C*	*A. thaliana*	PEG ABA NaCl	CaMV 35S	*Populus tremula × tremuloides*	NaCl	↑ abiotic stress tolerance↓ OEX did not induce gene expression in poplar as in *A. thaliana*	Yu X. et al., 2017
**Hydrolases superfamily protein***PAD4*	*Populus tremula* x *tremuloides*	SA drought UV-light root hypoxia	RNAi	*A.thaliana Populus tremula* x *tremuloides*	drought	↑ drought sensitivity ↓ water use	Szechynska-Hebda et al., 2016

*Overexpression (OEX), Polyethyleneglycol (PEG), 1-naphthaleneacetic acid (NAA), 6-benzyl aminopurine (6-BA), gamma-Aminobutyric acid (GABA), gibberellic acid (GA), abscisic acid (ABA), superoxide dismutase (SOD), ascorbate peroxidase (APX), Salicylic Acid (SA), peroxidase (POD), malondialdehyde (MDA), Cauliflower mosaic virus (CaMV), Electrolyte leakage (EL), ↑, Increase; ↓, Decrease*.

*Data were searched between 2013 and 2018 with the key words: Tree and overexpression and drought in Web of Science*.

In most cases, candidate genes for stress tolerance were expressed under the *35S* promoter, leading to high constitutive production in the transgenic plant (Table 1). A drawback of this approach is that more drought resistant plants often show biomass yield trade-off (e.g., the dwarfed *eui* mutant, a mutant in the GA-regulating *CYP714 A3* gene, Wang C. et al., 2016). The utilization of stress-inducible promoters may be promising to achieve a balance between growth under non-stress conditions and enhanced defense activation under drought conditions. For example, a novel zinc finger protein from the succulent, xerophytic species *Z. xanthoxylum* rendered transgenic plants more tolerant to osmotic stress (Chu et al., 2016; Table 1). Similarly, overexpression of *DREB* (dehydration responsive protein binding element) under the *RD29* promoter activated osmolytes (sugars) and enhanced the performance of transgenic plants under drought stress (Zhou et al., 2012; Table 1). Other studies showed successful activation of antioxidants, reduction of membrane leakage and increased photosynthesis, when *YUCCA6* (a flavin mono-oxygenase-like from *Arabidopsis thaliana*) or choline oxidase (from bacteria) were overexpressed under an oxidative stress-inducible promoter (Ke et al., 2015, 2016; Table 2). However, overall utilization of stress-inducible promoters is still rare.

Plant model species, in which drought responses have often been studied and for which genomic information is available for a decade or longer, such as *A. thaliana* and *Populus* spp. were often used as the source species of inducible genes. In recent years, the gene pool of drought and salt tolerant woody species has increasingly been tapped. Among these species are: the succulent, xerophyte *Z. xanthoxylum*, the salt-tolerant and facultative succulent poplar, *P. euphratica*, the salt- and drought-tolerant species, *Tamarix hispida* and the salt-tolerant *Fraxinus velutina* (Tables 1, 2). Other crops and woody species that have also been increasingly used as gene source are: *Diospyros kaki* (a widely cultivated fruit tree in China), *Phyllostachys edulis* (bamboo), *Morus* spp. (mulberry, feed for silkworms), *Hevea brasiliensis* (rubber), *Picea* and *Pinus* (conifers), and *Cicer arietium* (herbaceous legume crop such chikpea). The target species were model species such as poplars, *Arabidopsis* and *Nicotiana tabacum*, but also crops such as alfalfa, cotton, lotus, tomato and rice. Transformation of non-model tree species for enhanced stress tolerance is still rare but recent results showed promise. Overexpression of *DREB2A*, a gene that forms a hub for drought-stress related gene expression in *Robinia pseudoacacia* resulted in enhanced drought resistance (Xiu et al., 2016). The drought resistant phenotype was mediated by the formation of deeper roots and decreased oxidative stress, and most likely mediated by effects on the phytohormone balance of the plants (Xiu et al., 2016, Table 1).

Succulence, which occurs in many drought or salt resistant species, is a complex trait that may prove to be useful for drought resistance. Leaf thickness and water content increase with increasing salinity and aridity (Ottow et al., 2005; Nguyen et al., 2017). Succulent leaves exhibit a significant water storage capacity and dilute intrinsic salt concentrations (Ottow et al., 2005; Scholz et al., 2011; Han et al., 2013; Ishii et al., 2014). Overexpression of a putative xyloglucan endotransglucosylase/hydrolase from *P. euphratica* (*PeXTH*) contributed to salt-induced leaf succulence (Han et al., 2013) by improving cell wall properties to cope with water deficit and high salinity (Cho et al., 2006). Overexpression of a hot pepper (*Capsicum annuum*) *CaXTH3* in guard cells reduced transpiration under dehydration stress, thus, supporting a role of *XTHs* in drought resistance (Choi et al., 2011).

As highlighted before, the acclimatory responses of trees to drought invoke a multitude of molecular and biochemical changes. Consequently, a focus of many recent genetic approaches was on genes encoding protein kinases and transcription factors to potentially target whole signaling and biochemical pathways instead of single gene products. Overexpression of *CPK* (calcium-dependent protein kinase, Chen et al., 2013), *bZIP* (Basic leucine zipper protein, Wang et al., 2010), *DREB* (dehydration-responsive element-binding protein, Chen et al., 2009, 2011; Zhou et al., 2012; Yang et al., 2017), *CBL* (calcineurin B-like protein, Li et al., 2013), *NAC* [no apical meristem (NAM, Wang L. et al., 2016, 2017), *ATAF* (*Arabidopsis* transcription activation factor), *CUC* (cup-shaped cotyledon) superfamily, Wang et al., 2013; Lu et al., 2017], *ERF* (ethylene response factor, Wang et al., 2014; Yao et al., 2016), *WRKY* (Zheng et al., 2013), and *ZFP* (zinc finger proteins, Zang et al., 2015) often resulted in enhanced photosynthesis, higher WUE, higher activity of antioxidative enzymes, lower oxidative damage and improved growth under osmotic stress (Table 1). Examples are still rare where drought and salt responses are not congruent (Table 1). For example, overexpression of a *MAPK1* of the MAPK C family resulted in more salt tolerant but less drought and heat resistant plants but the underlying mechanisms for this difference are speculative (Liu et al., 2017).

ABA is crucial in mediating plant drought responses. Most of the signal transduction and response factors used for stress amelioration are regulated by ABA (Table 1). The receptor RCAR is the first target of ABA and forms a complex with PP2C for stress signaling (Fujita et al., 2011; de Zelicourt et al., 2016). The situation is even more complex because multiple RCARs and PP2Cs exist that are forming combinatorial interaction networks (Tischer et al., 2017). *Arabidopsis* and hybrid poplar overexpressing *RCARs* from *P. trichocarpa* were more drought tolerant than the wildtype through decreased water loss and increased osmotic and antioxidative protection (Yu J. et al., 2016, 2017). However, there is also fitness trade-off because germination of the overexpressed *Arabidopsis* seeds is inhibited (Yu J. et al., 2016). The transgenic poplar phenotype was normal and the biomass gain under strong drought stress was higher than that of the controls (Yu J. et al., 2017). HAB1 was suggested to be the ortholog of *Arabidopsis* PP2C, which is a negative regulator of ABA signaling and acts as co-receptor for RCARs. *Arabidopsis* overexpressing *HAB1* gene from *P. euphratica* lost ABA sensitivity and became more drought sensitive than the wildtype (Chen et al., 2015). Overall, functional characterization of these genes indicated that the core ABA signaling pathway is conserved in poplar and may be a suitable target for genetic engineering.

Studies applying novel gene editing methods (CRISPR/Cas9) to improve drought tolerance are still in their infancy but hold promise for new discoveries. For example, lignin deposition was reduced in poplars in which *Myb170* expression was abolished by CRISPR/Cas9 (Xu et al., 2017). Surprisingly, heterologous expression of *Myb170* in *Arabidopsis* uncovered its presence in guard cells, which showed stronger stomatal closure at night and thereby, enhanced drought protection (Xu et al., 2017). This study illustrates that novel functions of genes can be detected by combining CRISPR/Cas9 and overexpression.

## Conclusions and Research Needs

Trees are capable of responding to drought stress through a wide variety of cellular and physiological acclimation strategies, which form the basis for genetic improvements of drought tolerance. In particular, overexpression of drought sensing, signal transduction, and drought responsive transcription factors can enhance drought tolerance in a variety of model systems and some economically important woody species. Our overview on transgenic modifications revealed that modifications at the cellular level were the main targets, often using genes from drought or salt tolerant woody species for overexpression. However, systematic studies to clarify if these genes perform better than those from drought sensitive species are lacking. Comparative studies suggest that amplification of distinct gene families such as the SOS pathway in *P. euphratica*, gene duplication, and evolutionary recruitment of distinct metabolites such as ABA for stomatal regulation could also be important avenues for future research. Furthermore, long-term studies under field conditions are still scarce. There is obviously a strong need for testing genetically modified trees in their natural environment because the combination of stress factors such as heat and drought together may overrule the effects of single stressors present under laboratory conditions.

At a wider scale, we have to assert that our mechanistic understanding of the interplay among osmotic regulation, hydraulic adjustment and uptake systems for water and nutrients is still in its infancy. In particular, the root-to-shoot communication that sets off a suite of responses leading to morphological changes of the root system is not clear. Therefore, an important future task will be to uncover the genetic basis for an optimized resource allocation between biochemical defenses and production of new structures such as deep rooting systems under stressful climatic conditions. Next-generation genomics and phenomics approaches will facilitate a better understanding of phenotype-genotype maps and help to formulate genomic-assisted breeding strategies in forest trees for resistance to drought stress and other osmotic cues.

## Author Contributions

AP, SC, CE, and AH drafted and wrote the manuscript together. All authors agreed on the final version of this review.

### Conflict of Interest Statement

The authors declare that the research was conducted in the absence of any commercial or financial relationships that could be construed as a potential conflict of interest.
